# Starch-Directed Synthesis of Worm-Shaped Silica Microtubes

**DOI:** 10.3390/ma16072831

**Published:** 2023-04-02

**Authors:** Yang Chen, Michael A. Brook

**Affiliations:** Department of Chemistry and Chemical Biology, 1280 Main St. W, Hamilton, ON L8S 4M1, Canada

**Keywords:** silane-modified starch, silane-crosslinked starch, sol–gel synthesis, enzymatic degradation, worm morphology

## Abstract

Many strategies have been adopted to prepare silica materials with highly controlled structures, typically using sol–gel chemistry. Frequently, the alkoxysilanes used in sol–gel chemistry are based on monoalcohols, e.g., Si(OEt)_4_. The structural control over silica synthesis achieved by these precursors is highly sensitive to pH and solvency. Alkoxysilanes derived from the sugar alcohol glycerol (diglycerylsilane) react more slowly and with much less sensitivity to pH. We report that, in the presence of cooled aqueous starch solutions, glyceroxysilanes undergo transesterification with the sugars on starch, leading to (hollow) microtubules resembling worms of about 400 nm in diameter. The tubes arise from the pre-assembly of starch bundles, which occurs only well below room temperature. It is straightforward to treat the first-formed starch/silica composite with the enzyme amylase to, in a programmed fashion, increasingly expose porosity, including the worm morphology, while washing away untethered silica and digested starch to leave an open, highly porous materials. Sintering at 600 °C completely removes the starch silane moieties.

## 1. Introduction

An enormous literature, including the comprehensive book by Iler [1], exists around the preparation and properties of silica in its many forms. This is no surprise, as references indicate that the Earth’s crust comprises 70–80% silica (although it is easy to find references ranging from 12–95%) in one form or another, making it the planet’s dominant inorganic material. Given its wide availability, it is unsurprising that organisms including diatoms, radiolarians and sponges use dissolved silicic acid as a resource that, after suitable manipulation in nature, leads to the formation of the beautiful exoskeleta of these organisms [2].

Materials scientists have spent considerable time searching for synthetic strategies that permit the controlled assembly of siliceous structures for a variety of purposes that ranging from reinforcement provided by very small, essentially pore-free particles (often found as aggregates) of fumed silica to model colloidal systems formed via the Stöber process [3], which involves (simple?) hydrolysis and condensation of alkoxysilanes to yield nearly monodisperse spheres. This sol–gel approach is increasingly the most common and practical way to control the 3D structures of silica [4]. In addition to relatively inexpensive and available precursors, sol–gel silica processes benefit from various levels of structural control provided by concentration, pH and the presence of various salts. With appropriate surface-active materials, it is possible to introduce porosity with multiple levels of hierarchical control [5,6], allowing one to use similar chemical processes to target useful materials. Classic examples of this approach depend on the self-assembly of surfactants, including for zeolite synthesis (e.g., MCM41) [7], or nanotube structures [8], such as those formed by the boric acid crosslinking of surfactant micelles [9]. Sol–gel processes also permit the synthesis of simple [10] or double-walled [11] nanotubes. 

We are still far behind nature in our ability to control the structures of siliceous materials. It is possible to assemble silica in a controlled manner by ‘growing it’ on pre-existing structures (templating) using sol–gel processes, such as on cellulose acetate [12] or polystyrene fibers [13]. In some cases, the template is a contributor to the physical properties of the composite, as in the case where the mechanical properties of silica are enhanced by forming a composite with starch [14] or templating on electrospun silk [15]. In others, the template translates to function. For example, mesoporous silica grown on chiral polymers could be used as a chromatographic support for the separation of enantiomers [16]; entrained biological molecules can serve as immobilized enzymes [17], or to select drug targets using protein affinity [18]. Silica nanotubes grown on carbon have found use as anodes [19]. More often, however, having served to control assembly, the organic/biological template is removed by calcination to reveal the pure metal oxide framework, as in the elegant templating studies using agarose on which TiO_2_ and other metal oxides have been grown [20], or starch gels used to create zeolites [21].

More relevant to this work is the use of biological templates that, after silica synthesis, can be removed by biological degradation under mild conditions, for example, in protein-templated silica [22,23]. Polysaccharides have been widely used in combination with silica, including as templates [24]. Starch is a particularly useful template, as its enzymatic degradation is facile, for example, using the enzyme amylase.

**Figure 1:**
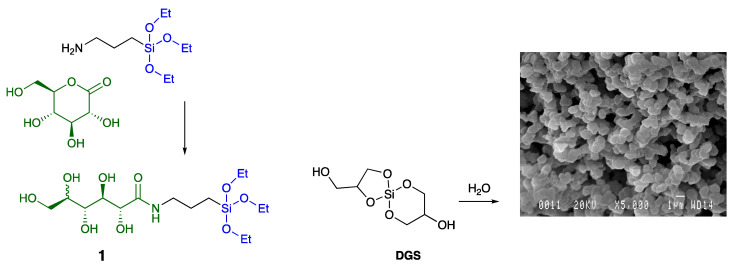
**DGS**, **1**, and the resulting monolithic silica formed (at **left**) by hydrolysis condensation.

The most commonly used silica precursor for sol–gel chemistry is TEOS (Si(OEt)_4_), or its more reactive analogue, TMOS (Si(OMe)_4_). The formation of silica—both rates of assembly and resulting structures—is highly dependent on pH, (co)solvents and reagent concentrations. Hüsing et al. [25] and we [26,27,28] have found great benefit in using alkoxysilanes derived from glycols. The glycol-containing starting materials and products: ethylene glycol; propylene glycol; glycerol; and others dramatically change both kinetics and the pH profile of cure chemistry when compared to TEOS. For example, in the case of glycerol, which was used to form diglycerylsilane starting material, **DGS** (diglycerylsilane), comparable gelation times were observed over a large pH range of ~6–11; rates of cure were easily retarded simply by adding additional polyol. **DGS** is also highly compatible even with sensitive proteins such as luciferase [29]. Its use as a silica starting material for silica monoliths was accompanied by significantly lower levels of shrinking (and cracking) than was the case for traditional alternatives. The biocompatibility, as judged by reduced protein denaturation, of silica structures prepared from **DGS** was further enhanced by the presence of the trialkoxysilane derived from glucose **1** in the sol–gel process (Figure 1) [30].

It was of interest to explore if, rather than entraining proteins or polysaccharides in silica, it would be possible to benefit from the properties of DGS while templating the polysaccharide starch. We report the formation of starch-templated silica and microscale worm-like morphology that were revealed once the starch was partly or completely digested by the enzyme amylase, but lost during calcination.

## 2. Materials and Methods

### 2.1. Materials

Soluble starch was purchased from BDH Inc. or Sigma Aldrich. α-Amylase from *Bacillus amyloliquefaciens* was purchased from Sigma. Imidazole, calcium chloride, tetraethyl orthosilicate and glycerol (99.5%, anhydrous) were purchased from Aldrich. All chemicals were used as received. Diglycerylsilane **DGS** was prepared according to published work [28,31].

### 2.2. Methods

^1^H, ^13^C and ^29^Si NMR spectra were recorded on a Bruker Avance 600 MHz nuclear magnetic resonance spectrometer using chloroform-*d*. A thermogravimetric analysis (TGA) was performed using a Thermowaage STA409. The analysis was measured in air, with a flow rate of 50 mL/min. The heating rate of 10 °C/min was used from room temperature to 900 °C. DSC were recorded on TA instruments DSC 2910 using standard DSC cell. SEM photographs were observed using JEOL JSM-7000F Field Emission Analytical Scanning Electron Microscopy.

Prior to N_2_ sorption measurements, all the samples were degassed at 100 °C under vacuum overnight. Nitrogen adsorption–desorption isotherms were recorded on a Quantochrome Nova 2000 instrument. The specific surface area was calculated using the multipoint Brunauer–Emmett–Teller (BET) method. Pore size distributions were calculated by the Barrett, Joyner and Halenda (BJH) method. Pore volumes were determined from the amount of N_2_ adsorbed at P/P_0_ = 0.99.

### 2.3. Preparation of Silica-Starch Gel

Starch solutions were freshly prepared by dissolving soluble starch in water at 90 °C for 10 min, at specified weight ratios of 30 and 40%, respectively, and kept warm (90 °C) before use.

Shown for entry **W1** (Table 1): **DGS** (0.40 g, 2.0 mmol) was dissolved in DI water (0.50 mL) using an ultrasonic bath for 20 min at 0 °C. The resulting sol was mixed with starch solution (starch 0.15 g and water 50 mL). The mixture was allowed to stand at 0 °C for 30 min then at −10 °C for 1 h, and allowed to warm to room temperature overnight, leaving gels that ranged from translucent to opaque **W1** (higher starch content materials were more opaque). It could be stored in a sealed container at this stage. The process was repeated with higher starch loading (0.20 g and water, 50 mL) to produce **W2**. Other ratios of DGS/starch/water were examined but did not lead to worm formation (Table 1). Note that the ability to create worm morphologies depends on the nature of the soluble starch. The starch obtained from Sigma Aldrich (Oakville, ON Canada)—using the same conditions—never produced worm-like moieties. Thus, when using starch from a different supplier, it will likely be necessary to optimize the process to facilitate both the starch self-assembly and concomitant sol–gel process. Prior to calcination or any measurement of SEM, the samples were washed with water (see next section) and lyophilized. Calcination was performed at 600 °C in a Barnstead Thermolyne Furnace 1400 FB1415M at room pressure for 5 h.

### 2.4. Digestion of Starch with Amylase

#### 2.4.1. First Digestion

**Figure 2:**
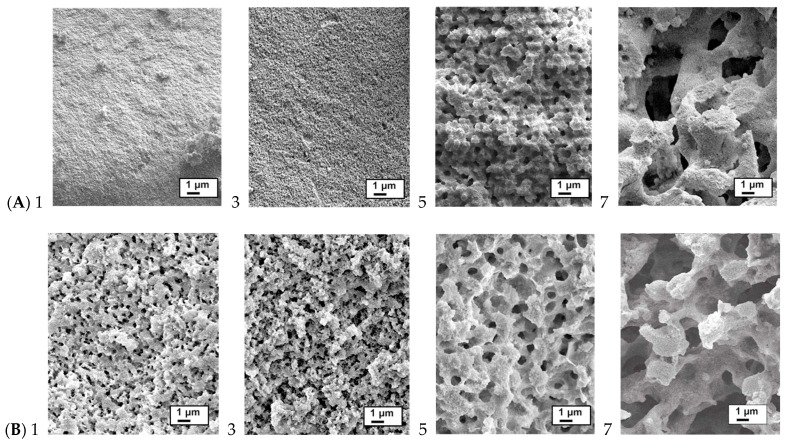
As synthesized silica (entries Table 1) (**A**) after calcination at 600 °C and (**B**) after 1 digestion using amylase (SEM micrographs were taken after lyophilization). The numbers shown 1–7 reflect the samples, as listed in Table 2.

The resulting wax-like gel was soaked in DI H_2_O (10 mL) for 4 h, a process that was repeated 9 times over 2 d, each time with fresh water. **W1** and **W2** were incubated in imidazole-HCl buffer solution (1.0 mL, 50 mM, pH 6.5, containing 2.0 mM of CaCl_2_) containing α-amylase (0.2 g) at 40 °C for 20 h, washed by soaking in H_2_O (10 mL) for 4 h and repeated 5 times. The gel was then lyophilized (dried under vacuum) to give **W1-A1** and **W2-A1**. Other samples were similarly exposed to these conditions, but worms were not revealed after digestion (Figure 2 and Figure S1, Table 2). Erosion was demonstrated to occur ‘from the outside in’ by placing a sample in the enzyme solution, removing it after 8 h, washing repeatedly with water, lyophilizing and then cutting the sample in half to allow an examination of the cross-section by SEM.

#### 2.4.2. Second Digestion

**W1-A1** and **W2-A1** were incubated in imidazole-HCl buffer (1.0 mL, pH 6.5) solution containing α-amylase (0.2 g) at 50 °C for 20 h, washed by soaking in H_2_O (10 mL) for 4 h, a process that was repeated 5 times. The gel was then allowed to dry in air to produce **W1-A2** and **W2-A2**, respectively.

## 3. Results

Silicon-containing starch was simply prepared by mixing a warm solution of dissolved starch in water with **DGS**. Note that, even with soluble starch, the specific batch and supplier can be important for morphologies of the resulting starch silica gels. After mixing with mechanical stirring, the solutions were cooled first to 0 °C and then −10 °C and left overnight at room temperature. The samples were repeatedly washed with water to remove glycerol and unreacted **DGS** and then lyophilized. A library of starch/silica composites was prepared, in which the relative concentrations of both **DGS** and starch were varied (Table 1). The samples were calcined at 600 °C to remove all organic constituents. It quickly became apparent that the degree to which microporous materials formed was highly dependent on this ratio; higher starch ratios led to much more open structures (Figure 2A).

**Figure 3:**
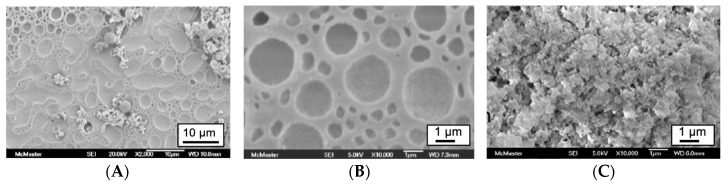
As-prepared (**A**) upper gel surface from recipe 6 (Table 1) (scale bar 10 microns); (**B**) higher magnification of A (scale bar 1 micron); (**C**) the same sample calcined at 600 °C 1200–1400 m^2^ g^−1^ (see also Figure S1).

The surfaces of the gels presented unusual features that, we at least, have not previously observed (Figure 3). The materials exhibit a multiphase surface. Naively, we initially assumed that the water soluble entities, **DGS** and starch, would readily mix. Initially they do, but as silica formation occurs, two phases evolve that have different domain sizes. If one calcines these (or any of the starch/silica composites) most of these features are lost, consistent with an interface that is partly organic and, upon thermal stress, is able to undergo further metathesis/condensation reactions as the organic constituents are lost, leaving only silica behind (Figure 3A,B vs. Figure 3C).

**Figure 4:**
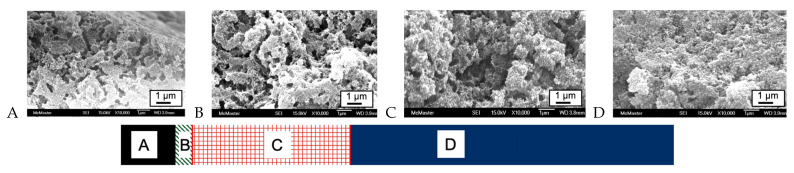
Cross-section of a starch/silica sample after enzymatic digestion; the largest changes were observed in domain C. The sample was ~16 mm^2^ × 2.2 mm thick. SEMs were taken at depths of (**A**) 5 μm; (**B**) 275 μm; (**C**) 650 μm; (**D**) 1.1 mm. All SEMs were measured at 10,000× and the scale bar shows 1 mm.

The ability to selectively remove the starch under mild conditions at room temperatures, rather than by calcination, was tested. Rather than lyophilizing the starch/silica materials, they were subjected to sequential enzymatic degradation of the starch contained within the sample. It quickly became apparent that amylase ‘ate its way’ from the outside to the core. A cross section from the top to the bottom of a given sample showed four domains: (A) the outer, air-exposed layer that, while porous, did not undergo significant changes during enzymatic degradation; (B) a narrow band exhibiting slightly higher porosity, (C) a large domain where significant quantities of starch had been removed and the porosity was increased; and (D) the unmodified starch silica (Figure 4). The impact of starch content on porosity after enzymatic degradation, rather than calcination, is seen in Figure 2.

**Figure 5:**
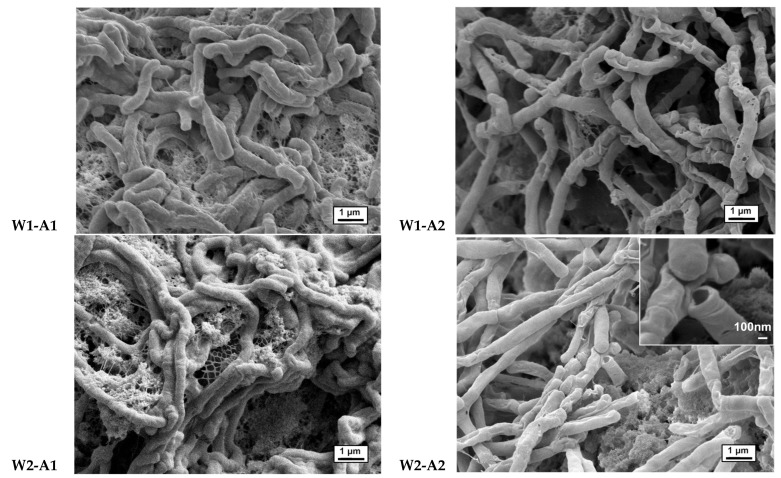
SEM images of worm-shaped microtubes after (**left**) the first amylase digestion **W1-A1, W2-A1** and (**right**) second amylase digestion **W1-A2**, **W2-A2**. Scale bar, 1 μm (inset for **W2-A2** at higher magnification, scale bar 100 nm).

In selected samples, worm-shaped morphologies were exposed upon the partial removal of starch by amylase digestion at a lower temperature of 40 °C; secondary enzymatic treatment of these samples at 50 °C led to even more open structures with a higher fraction of ‘worms’ and lower quantities of background material. That is, the background matrix of starch and any silica or small elements are removed during amylase digestion, but the tubes/worms are not subject to enzymatic degradation. SEM images reveal that the starch–silane microtubes are highly uniform and about 400 nm in diameter. They appeared curving and entangled on the surface of porous matrix, suggesting some level of flexibility (Figure 5); many appear hollow. As noted above, the tube morphologies completely disappear upon calcination at 600 °C (performed using a slow temperature gradient of 10 °C min^−1^, Figure 3C).

We note that two factors are required if the worm-like morphologies are to form within the silica matrix: starch/ratio; and temperature. First, the influence of the silica/starch ratio on the formation of worm-shape microtubes was investigated at different Si/starch (mmol/mass (g)) ratios of 2:0.1 ➞ 2:0.4 (Table 1). However, the formation of worm-shaped microtubes (the tubes were only hollow after enzyme digestion; otherwise, they could be considered wires) was only (reproducibly) obtained in two ratios of 2:0.15 and 2:0.2. In these two cases, the diameters of the microtubes were not affected by the initial ratio of starch in the system (Figure 5, **W1** vs. **W2** samples). A much higher yield of microtubes was obtained in the ratio of 2:0.15. Second, after mixing the starch and **DGS**, it is necessary to cool the mixture below 0 °C and allow the samples to anneal. Only when both of these conditions were met were the worms observed.

**Figure 6:**
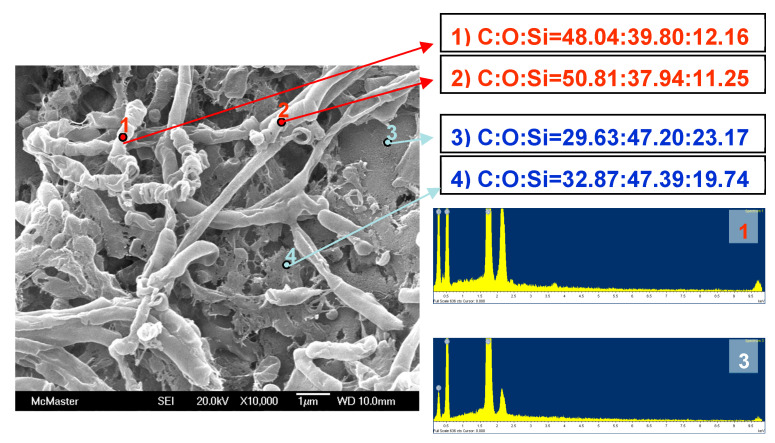
EDX analyses of worms and the background matrix. The numbers in the labels (**right**) show atomic percent ratios.

**Figure 7:**
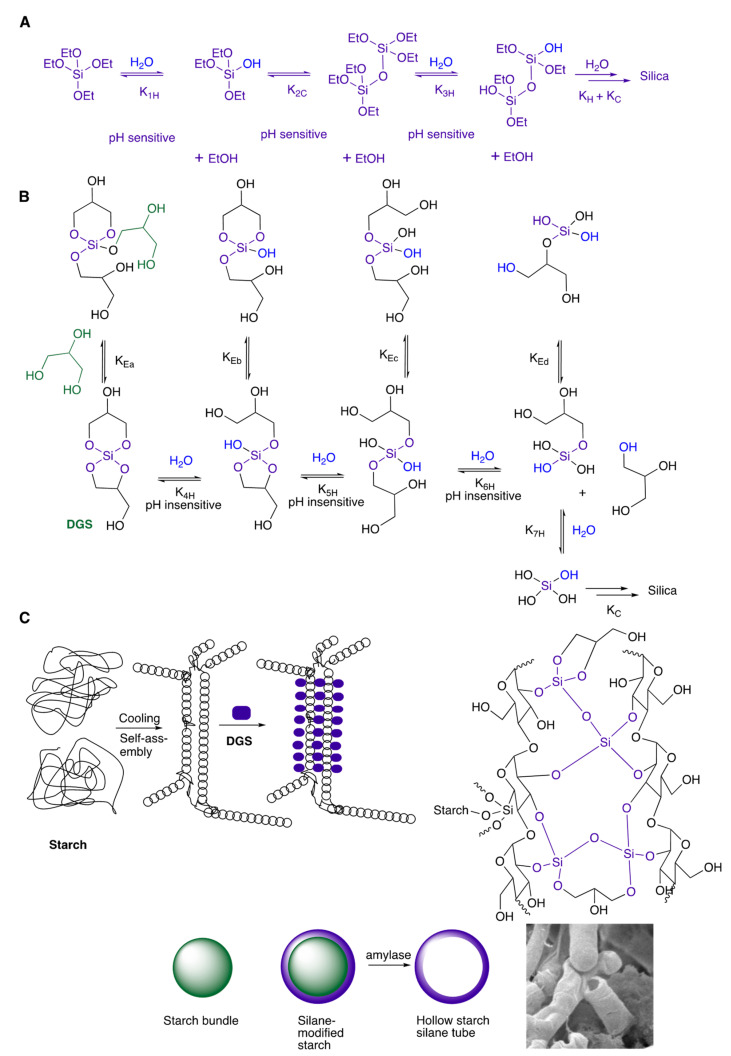
Model reactions leading to silica/starch composites from (**A**) TEOS, (**B**) DGS and (**C**) transesterification with starch (adapted from ref. [20]).

The nature of the worm materials was probed using an EDX analysis (Figure 6). A comparison of the elemental composition of the ‘worm’ vs the background material shows a much higher carbon atom% on the worm. Note: **DGS** has a C:O:Si ratio of 36.7:48.9:14.3 and starch a C:O ratio of 48.7:51.3. Thus, a small fraction of silicon is proposed to bridge starch assemblies to create these worms. A ‘model’ structure—which is entirely speculative—that has the correct elemental ratio is shown in Figure 7.

**Figure 8:**
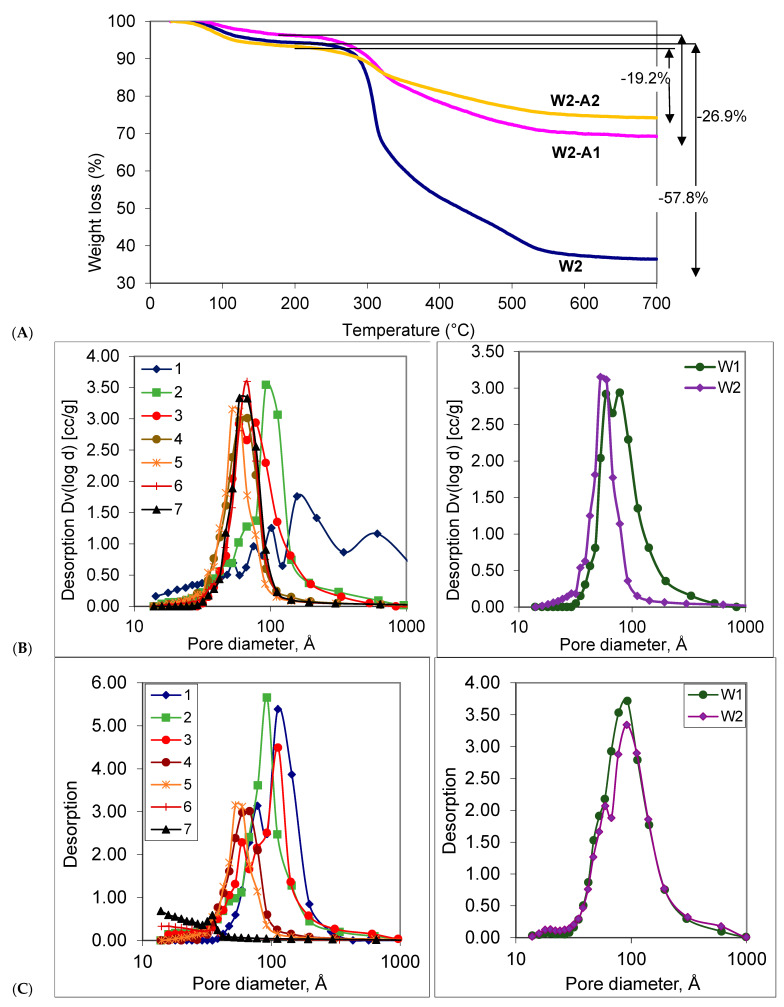
(**A**) TGA analyses of **W2**, **W2-A1** and **W2-A2**. (**B**) Pore size distribution of the samples after (**B**) 1st enzyme digestion and (**C**) after calcination at 600 °C of typical samples (**left**) and worm-containing samples (**right**).

TGA analyses of the worm-containing samples are summarized in Figure 8. All samples exhibited two types of weight loss. Water was lost from all samples as they were heated from room temperature to about 150 °C: the percentage of weight loss due to water was −5.9% for as-prepared sample **W2**; −4.7% for **W2-A1**; and –6.5% for **W2-A2**. The second mass loss greatly differentiated samples that had undergone partial or complete enzymatic digestion. The as-prepared materials showed further weight loss of −57.8% (**W2**), while lower losses were observed for once- −26.87% (**W2-A1**) and twice-digested −19.2% (**W2-A2**) samples between about 150 and 600 °C. This loss is ascribed to starch carbonization and combustion, as could be seen from the TGA data of starch gel alone, which showed 100% mass loss by 600 °C. That is, oxidizable carbon is removed from these samples by enzymatic digestion prior to the TGA measurement.

Nitrogen absorption data revealed Type IV isotherms with a single hysteresis loop for all samples. BJH pore size distributions show that almost no micropores smaller than 2 nm are present (Figure 8B,C). With the exception of sample 1 (Table 1), after the removal of starch by amylase digestion, the samples possessed a relatively narrow pore size distribution. Samples containing a higher fraction of starch in the formulation exhibited a lower total surface area and in pore volumes (Table 2); average pore diameters decreased with increasing starch. Calcination typically led to increases in surface areas and total pore volume, while average pore sizes slightly decreased.

## 4. Discussion

The sol–gel process for silica formation involves a series of hydrolysis and condensation steps. When alkoxysilanes undergo hydrolysis (a series of K_H_s), the process is typically ‘one-way’. The equilibrium lies very far towards the product silanol (i.e., K_1H_ and K_3H_ are large, Figure 7A) and then condensed silica; the process is very pH sensitive [1]. By contrast, glycol-derived alkoxysilanes exhibit more complex equilibria [26,27,28]. In addition to the sequential hydrolysis and condensation shown with TEOS, the initially formed silanols participate in a series of unproductive equilibria that involve exchange at silicon but no net changes in bonding (K_Ea-d_, Figure 7B). The processes are consequently less sensitive to pH. While the relative magnitude of the K for TEOS vs **DGS** is unknown, the rates at which the equilibrium is reached are lower for glycolsilanes.

In addition to the hydrolysis/condensation and K_E_ shown, the glycol silicanes and intermediates can undergo transesterification with other glycols. We hypothesize that the transesterification of these intermediates with starch is key to the observed formation of worm morphologies. In analogy with **DGS** itself, starch siloxanes will undergo very slow hydrolysis because of competition transesterification processes with the multitude of available alcohols once the silane is so tethered. That is, in the absence of the continuous introduction of water and loss of silanol (or starch) groups, complete hydrolysis is essentially paralyzed.

Within the starch/silica composites formed are three types of materials: unmodified starch, untethered silica (possibly modified with alkoxy groups) and silane-modified starch. Digestion with amylase degrades the starch, leading to an increase in porosity per se and a loss of silica that has not been integrated into the monolithic structure. Thus, materials created with higher initial starch loading became more porous upon enzymatic degradation, as there was more material to be lost (Figure 2B and Figure S1). Sequential amylase treatments under more vigorous conditions (50 °C) led to more open structures (Figure 5); however, not all the starch was amenable to degradation. One attractive feature of the process is that, starting from an initial starch/**DGS** formulation, one may decide what final porosity is desired and tune the amount of enzymatic degradation to perform accordingly.

With traditional porogens such as poly(ethylene oxide), the porosity of silica monoliths is controlled through a combination of sol–gel processes generating silica, and concurrent microscopic/macroscopic phase separation. After removal of the water-soluble PEO phases by washing or sintering, the remaining hierarchical porous structures often contain micro, meso and macropores [26,32]. 

In silica–starch systems, there are almost no micropores smaller than 2 nm present after either amylase digestion or sintering, which suggests that the sizes of starch gel that acts as a template are larger than 2 nm. PEO, of course, is water soluble and does not have such geometric constraints. Similar to silica-PEO systems, there is no direct relationship between mesopore sizes and starch gel fraction. The increase in pore volume that accompanied more starch in the starting formation is most likely due to a change from particle aggregate to bi-continuous porous structures that can be seen in the SEM images (Figure 2). With amylase—48–49 kDa, *R*_min_ (minimum radius) of about 2.4 nm—there is a physical limitation for its ability to access all starch-occupied pores. This inaccessibility accounts for the ~19% starch after digestion that remains in pores that are too small for the enzyme to access.

Worms were only observed in selected samples. This is attributed to subtle changes in the kinetics of the processes that occurred. The sol–gel synthesis of silica—already complicated even with simple molecules such as Si(OEt)_4_ (Figure 7A)—is more complicated when the additional transesterification processes with glycerol and with starch are considered (Figure 7B). In addition, the specific nature of the starch self-assembly that takes place upon cooling will depend on the specific starch used, its concentration in the formulation and the temperatures to which the formulation is exposed during silica synthesis. Small changes in these parameters can lead to samples where silane transesterification on starch bundles (Figure 7C) is less effective, and worm structures were not observed. We note that the processes here to create **W1**➞**W1-A2**, **W2**➞**W2-A2** were highly reproducible.

The worm moieties are neither silica nor starch but a composite of the two. The elemental analysis suggests that, after untethered starch was enzymatically removed, the resulting worms possessed more silicon than starch but much less than silica (Figure 6). We propose a partly crosslinked starch structure that is the consequence of glycol transesterification at silicon. The resulting product resists both hydrolysis and enzymatic degradation by amylase.

Starch is a polysaccharide containing branch amylopectin and linear amylose; the latter is composed of D-glucose repeat units in (α1→4) linkages. Soluble starch contains a high percentage of linear amylose, which governs its properties [33]. We infer that worms arise first from self-assembly of the starch /amylose upon cooling, as eloquently described by Caruso et al. [20] and others [34], and transesterification with silanes, which is expected to be a slow process at these temperatures. Over time, glycerol is partly or completely displaced by starch to give a starch–silica matrix embedded with worms (Figure 7C). This suggestion is supported by the observation of hollow worms (inset **W2-A2**, Figure 5). Only the starch that has undergone transesterification—at the periphery of starch bundles (or is completely sequestered within silica)—is resistant to enzyme treatment. The starch/silane structure of the worm, like starch itself, is fragile when compared to silica. Heating (e.g., calcination Figure 2A, Figure 8) shows complete loss of the starch constituents and structural modification that leads to less porous silica than the starch containing precursor.

There are several attractive features of this process to make structured organomodified silica: the starting materials are readily available and very inexpensive; the process is synthetically trivial; and one can tune the porosity of the silica composite using initial formulation, particularly starch content, and the degree of thermal degradation of the starch constituent. Perhaps most attractive, however, is the ability to control the morphology, including porosity of the monolithic structures, by using different enzymatic degradation conditions; materials with different porosity and density can be formed, including worms. In addition, the starting materials, starch and **DGS**, and product, are very rich in natural materials, which is one of the objectives of more sustainable materials [35].

## Figures and Tables

**Table 1:** Sample preparation conditions.

	Sample	DGS (g)/H_2_O (mL) ^(a)^	Starch (g)/H_2_O (mL)	Total H_2_O (mL)	Si/Starch/H_2_O mmol/g/g
1	SS-20-1-10	0.41/0.5	0.10/0.5	1.0	2/0.10/1.0
2	SS-20-1.25-10	0.41/0.5	0.125/0.5	1.0	2/0.125/1.0
3	SS-20-1.75-10	0.41/0.5	0.175/0.5	1.0	2/0.175/1.0
4	SS-20-2-15	0.41/0.5	0.20/1.0	1.5	2/0.20/1.5
5	SS-20-3-15	0.41/0.5	0.30/1.0	1.5	2/0.30/1.5
6	SS-20-3.5-15	0.41/0.5	0.35/1.0	1.5	2/0.35/1.5
7	SS-20-4-15	0.41/0.5	0.40/1.0	1.5	2/0.40/1.5
Worm	formulations				
**W1** ^(a)^	SS-20-1.5-10	0.41/0.5	0.15/0.5	1.0	2/0.15/1.0
**W2** ^(a)^	SS-20-2-10	0.41/0.5	0.20/0.5	1.0	2/0.2/1.0

^(a)^ The only samples that reliably led to worm structures

**Table 2:** Summary of surface area and total pore volume measured by N_2_ absorption after 1st enzyme digestion and after further calcination at 600 °C.

Sample after 1st Enzyme Digestion	Surface Area (m^2^ g^−1^)	Total Pore Volume (cc g^−1^)	Average Pore Diameter (Å)
1	519.3 ±11.2	1.616 ±0.13	124.5
2	412.3 ± 10.1	1.105 ± 0.10	107.2
3	405.0 ± 7.9	1.084 ± 0.11	107.0
4	412.3 ± 12.5	0.8432 ± 0.1	81.80
5	397.8 ± 7.1	0.7420 ± 0.08	74.60
6	358.9 ± 10.2	0.7794 ± 0.08	86.86
7	348.0	0.8079	92.86
**W1**	539.1 ± 10.2	1.602 ± 0.12	118.86
**W2**	498.2	1.658	133.12
Sample (after calcination at 600 °C)	Surface area (m^2^/g)	Total pore volume (cc/g)	Average pore diameter (Å)
1	551.0 ± 11.1	1.575 ± 0.12	114.3
2	655.2	1.562	95.34
3	614.0	1.497	97.51
4	556.3	1.065	76.58
5	612.5	1.012	66.10
6	539.8 ± 10.5	0.3298 ± 0.02	24.44
7	730.5	0.4474	24.49
**W1**	701.2	1.695	96.69
**W2**	641.1	1.577	98.39

## Data Availability

The data presented in this study are available in the supplementary material.

## Supplementary Materials

The following supporting information can be downloaded at: https://www.mdpi.com/article/10.3390/ma16072831/s1, Figure S1: SEM images of samples after enzymatic digestion and calcination.
