# Supplementary material for: Starch-Directed Synthesis of Worm-Shaped Silica Microtubes
Source: Materials (Basel). 2023 Apr 2;16(7):2831. doi: 10.3390/ma16072831 (PMC10096145; doi:10.3390/ma16072831)
Supplement: Supplementary file 1 [file materials-16-02831-s001.zip › materials-2277673-supplementary.pdf]

materials

MDPI

Supplementary Materials

Starch-directed Synthesis of Worm-shaped Silica Microtubes

Sample	After 1st enzyme digestion	After calcination at 600 °C
1	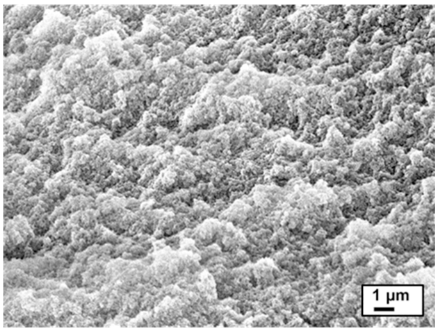	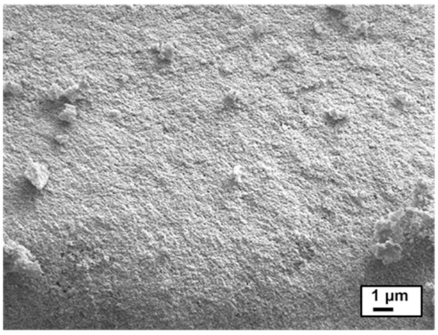
2	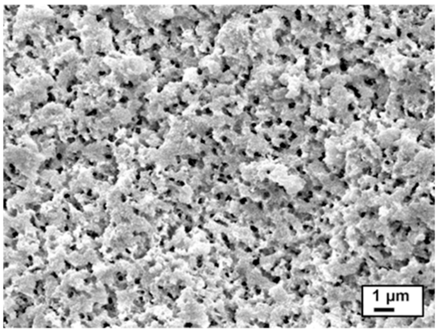	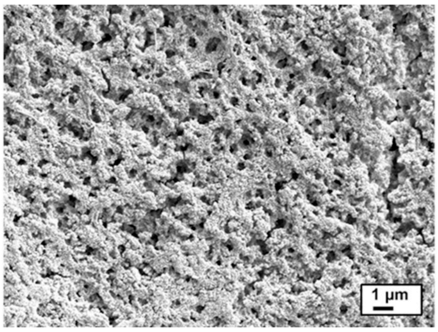
3	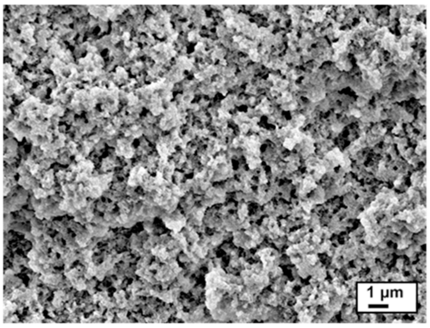	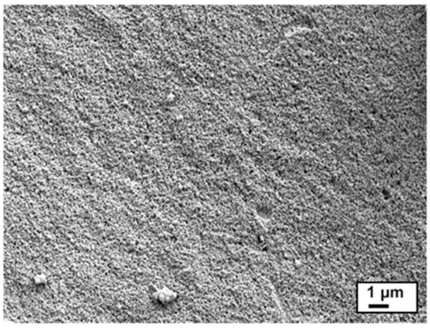
4	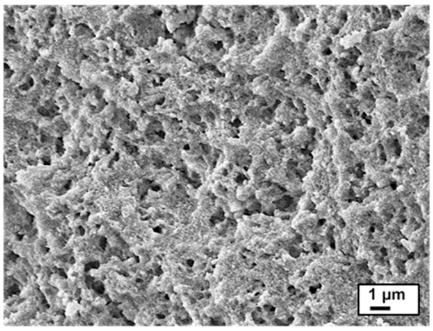	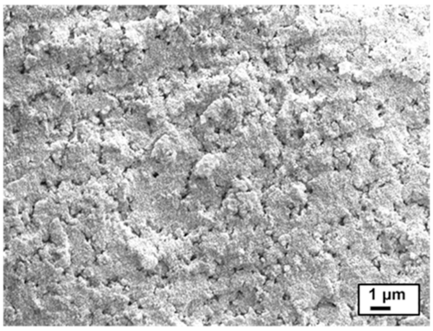

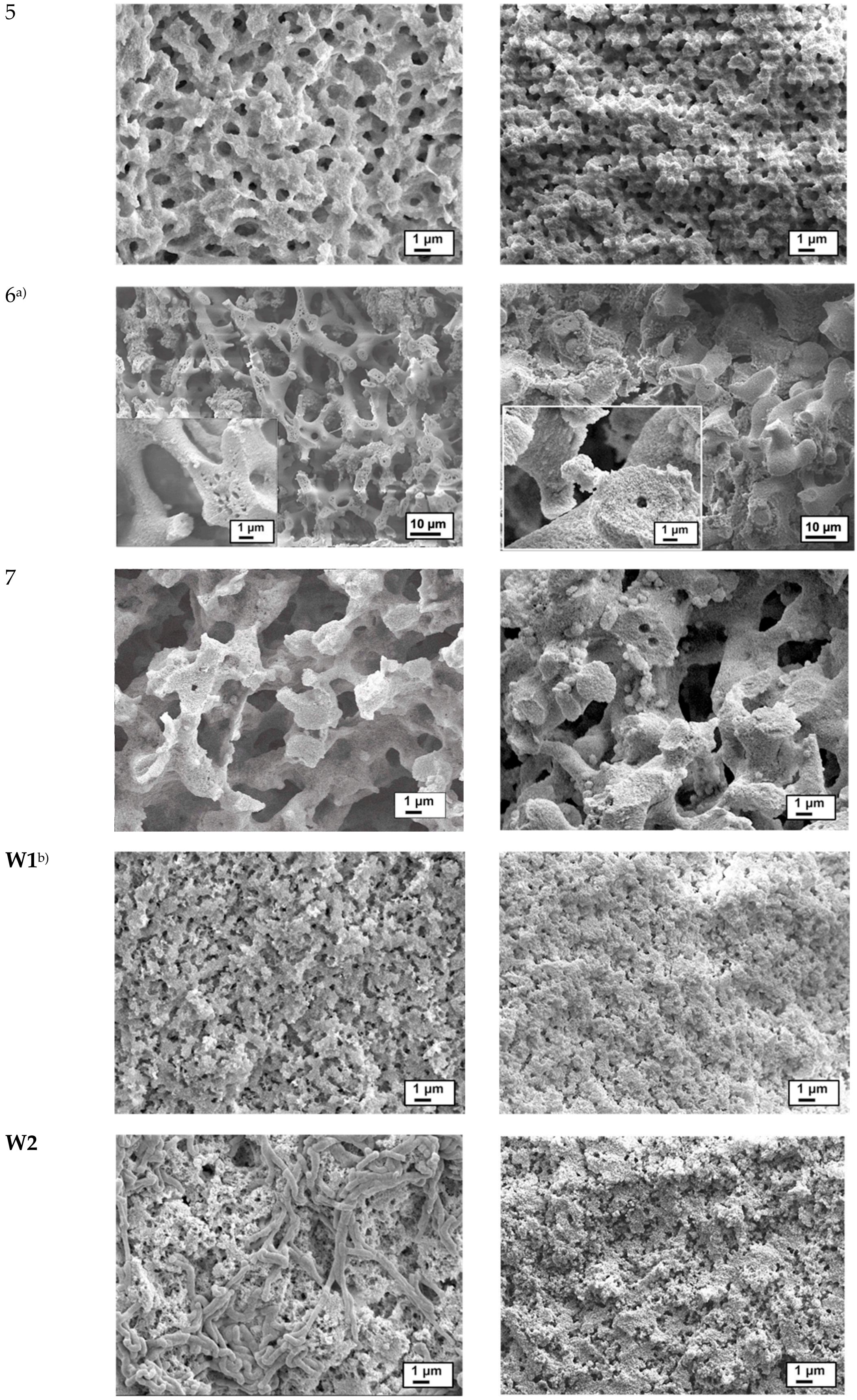

Figure S1. SEM images of the samples after 1st enzyme digestion (left) and after further calcination (right), magnification ×5000. a) The SEM image of sample 6 is at a lower resolution than the other

images, except for the inset image. [b)] The SEM image of sample **W1** was taken in an area of the sample that did not exhibit worm structures.
